# Clinical Considerations in Neurosurgical Radiosurgery in the Time of COVID-19

**DOI:** 10.7759/cureus.7671

**Published:** 2020-04-14

**Authors:** Susan C Pannullo, Swathi Chidambaram, Andrew Brandmaier, Jonathan Knisely, John R. Adler

**Affiliations:** 1 Neurosurgery, NewYork-Presbyterian/Weill Cornell Medical Center, New York, USA; 2 Neurosurgery, Weill Cornell Medical College, New York, USA; 3 Radiation Oncology, NewYork Presbyterian/Weill Cornell Medical Center, New York, USA; 4 Radiation Oncology, Stanford University Medical Center, Stanford, USA; 5 Department of Neurosurgery, Stanford University School of Medicine, Stanford, USA

**Keywords:** radiosurgery, neurosurgery, covid-19

## Abstract

The COVID-19 pandemic is affecting all aspects of the healthcare ecosystem, including administration of stereotactic radiosurgery (SRS). The clinical and logistical challenges created by the COVID-19 public health crisis are clear, but the solutions to these issues are less readily apparent. The goal of this work is to use our experience at a large, academic medical center as a lens for interpreting the many looming issues specific to radiosurgery and its role in the treatment of brain and spine disorders. While the full impact of the pandemic remains to be seen, the aim of this paper is to provide a structural framework to optimize delivery of neurosurgically oriented radiosurgery with proposed clinical workflow strategies. Innovative solutions to the current pandemic crisis affecting the healthcare ecosystem will be driven by increased interdisciplinary and global dialogue.

## Introduction

A complex and evolving situation

We are in the midst of a COVID-19 virus (“novel corona virus”)-related health crisis that is dramatically affecting medical practice around the world, particularly in large metropolitan areas such as New York City. As of April 8, 2020, 77,967 cases of COVID-19 have been identified in New York City with 3,602 deaths [1]. The rapid surge of cases poses not only a critical public health challenge but also presents significant medical and ethical implications for care of patients undergoing radiosurgery.

Currently, there is a paucity of literature guiding radiosurgery practice adjustments during an event like the COVID-19 pandemic. Although guidelines have recently been published regarding a triage algorithm for neurosurgical cases in the setting of COVID-19, no guidelines exist on how to balance the risks and logistical challenges associated with optimal and timely delivery of radiosurgery during this crisis [2-4]. The goal of this communication is to address some of the issues specific to radiosurgery and its role in the treatment of brain and spine disorders. While the full impact of the pandemic remains to be seen, the aim of this report is to provide a structural framework to optimize delivery of neurosurgically oriented radiosurgery with proposed clinical workflow strategies.

## Technical report

Current challenges and issues

Hospitals in New York City, like many others in the US, have mandated postponement of all elective surgical cases. The definition of “elective” remains problematic. In brain and spine open surgery, indications for emergent and urgent procedures are relatively clear. Radiosurgery is often considered to be an elective procedure. However, aspects of radiosurgery such as its minimally/non-invasive nature, outpatient setting, and reduced physical exposure compared with some other neurointerventions make it an important option at this time. An understanding of the unique role that radiosurgery can play in patient care is crucial when attempting to tackle the current challenges posed by the COVID-19 pandemic and when identifying potential strategies for practice modifications.

Radiosurgery is a multidisciplinary practice involving radiation oncologists, neurosurgeons, physicists, and radiation therapists, with crucial supporting roles played by neuroradiologists and nursing staff. Many high-volume radiosurgery programs use case review conferences to review cases, triage proposed radiosurgery procedures, and optimize treatments to minimize risks to patients and providers. In the past, such conferences were done in person. The need to practice social distancing has prompted a move to online platforms for these conferences. Fortunately, the nature of radiosurgery case review allows easy adaptation in most centers to continue these conferences remotely.

Following the trend of the remote conferences, and as with many clinical interactions now, telemedicine is increasingly replacing conventional office visits. Televisits via telemedicine lessen (or perhaps eliminate) in person consults and follow-up visits for radiosurgical care and limit the flow of traffic through hospital and outpatient office spaces. Thus, whenever possible, remote communication methods are being used within our radiosurgery practice. High-quality imaging for evaluation and treatment planning remain necessary for radiosurgery and surveillance of patients after a radiosurgical procedure cannot be forgotten, and for now remain available at our center and others.

In addition to the incorporation of social distancing practices within our radiosurgery workflow, our need to minimize exposure of patients and staff to the hospital environment has led to modification of our usual and preferred imaging surveillance frequency for patients being monitored before and after treatment. Scans are being deferred for patients with non-urgent benign conditions such as schwannomas or meningiomas. The radiosurgical treatment of these same patient cohorts is being temporarily postponed while public health agencies continue to report escalating spread of COVID-19. Surveillance imaging and treatments for these patients will resume when the multidisciplinary care team, in conjunction with hospital leadership, have determined that elective procedures can resume. At this point, for example, in patients with brain metastases whose cancer is well-controlled on systemic therapy and who are being monitored for intracranial tumor control, we are recommending an increase in inter-scan intervals from every two to every three months. In doing so, visit frequency will be reduced while still remaining within the range of the National Comprehensive Cancer Network (NCCN) consensus guidelines for routine surveillance of brain metastases [5]. These modifications may help decrease the risk of COVID-19 transmission and decrease non-COVID-19 related demands on the healthcare infrastructure.

As the number of COVID-19 cases continues to surge in New York City, there is growing concern that the magnitude of critically ill patients may overwhelm the healthcare infrastructure. The predicted extraordinary and sustained demands on our health systems are creating shortages in critical medical supplies such as personal protective equipment (PPE). Open surgery by nature requires more PPE than radiosurgery both during and after surgery. However, shortages of PPE may impact radiosurgery practice as well, since treatment still requires contact between patients and staff, placing both parties at risk for spreading or contracting infection. Similarly, providers in a radiosurgery program are at risk of exposure to patients who are themselves covertly infected with COVID-19.

A particular challenge in New York City has been lack of access to the N95 respirators needed to optimize the protection of radiation therapists who are directly in contact with patients during mask fabrication for computerized tomography (CT) simulation and treatment setup on the linear accelerator [6]. We strongly advocated for provision of N95 respirators and PPE for radiation therapists and other providers in our radiosurgery program appropriate to their level of exposure. The relative conservation of PPE in radiosurgery as compared to open surgery is an argument for greater use of radiosurgery, when appropriate, while overall healthcare access is compromised by the stresses introduced by the COVID-19 pandemic. For patients with malignant tumors in whom both open surgery and radiosurgery are credible options, the present scenario would favor radiosurgery.

A second tool for mitigating risk of exposure to providers and patients is COVID-19 testing. At a minimum, screening patients for fevers should be strictly enforced, not necessarily for therapeutic triage but to perhaps make staff more vigilant in their attention to hygiene and protection. In some settings, testing is being done upon entry to units or embarking on a course of treatment, in an effort to allow identification of infected patients. This testing, being conducted to minimize risk of staff-staff exposure to COVID-19 infected patients, will require mandatory self-quarantine and reduce staffing levels to potentially unsafe levels. Currently, wide-scale testing in the United States is infeasible due to a paucity of COVID-19 test kits. As more test kits become available, it may be possible to perform point of presence testing prior to the initiation of radiosurgery, although one could argue that currently employed universal precautions should be utilized irrespective of test results.

Redistribution of the medical workforce and reappropriation of hospital space are actively being carried out in anticipation of and in reaction to the demands of COVID-19 infected patients requiring hospitalization. It is possible that these actions may produce deficiencies in access to radiosurgery specialists and radiosurgery facilities. This would require consideration of how to continue providing access to radiosurgery, especially for those patients requiring treatment of intracranial metastases.

In many centers, the radiosurgery unit is a relatively open architecture, allowing family members to accompany patients into the treatment area. The amount of foot traffic is of new concern during this time of high risk of transmission of disease, and restriction of access of individuals not critical to the successful and prompt completion of the radiosurgical procedure should become routine during the pandemic. At our academic medical center, access for outpatient clinic has been restricted to the individual patient coming for evaluation unless there are mobility or language issues that preclude a solo visit.

An important indication for radiosurgery is brain metastases. Often cancer patients are immunocompromised, due to chemotherapy and their underlying disease. Treatment changes post radiosurgery can prompt prescription of dexamethasone or other corticosteroid medications, which can worsen immune suppression and therefore possibly become problematic for patients with COVID-19 infection. Careful consideration of the risk-benefit ratio of steroid therapy in patients at risk for COVID-19 associated infection is warranted, given the known immune suppressive effects of steroids.

## Discussion

Proposed clinical and workflow solutions

The clinical and logistical challenges created by the COVID-19 public health crisis are clear, but the solutions to these issues are less readily apparent. A meaningful strategy to finding robust solutions to these novel problems is to learn from the COVID-19 cases and challenges in other countries [4,7]. Until that evidence is available it would appear logical to ground the current practice of radiosurgery in the most widely accepted principles.

1. Develop agreed upon definitions of what is considered to be an urgent case for stereotactic radiosurgery. Conservative indications might include: symptomatic or enlarging brain metastases and spine metastases with proximity to spinal cord/conus/cauda equina) or producing intractable pain. More liberal indications would extend treatment to patients who were not symptomatic, and who did not have a systemic, targeted therapeutic option based on their cancer’s genomic profiling. It is likely that patients with ALK+ or EGFR mutation+ lung cancer or breast cancer brain metastases that are hormone or HER2 responsive and who are deemed highly likely to have a response to systemic therapy could have radiosurgical treatment deferred without an impact on quality of life or survival probabilities. Other extenuating circumstances such as projected loss of access to medical care should be considered.

2. Practice social distancing for the radiosurgical team.

a. Convert in-person multidisciplinary conferences to remote video meetings. In an effort to follow rules of social distancing, several measures have been taken to minimize contact among our radiosurgery team members. For instance, our weekly multi-disciplinary conferences have been transitioned to remote video conferences to minimize the number of people meeting in one room. Moreover, every effort is being taken to communicate remotely via emails and phone calls between team member and with patients in an effort to reduce contact and thus spread of infection. In many cases, these practice changes have improved the efficiency of our communication and cohesion of team members.

b. Use remotely accessible or web-based platforms for treatment planning. This will minimize need for direct interpersonal contact between neurosurgeons, radiation oncologists and the physics team.

3. Protect patients and providers from exposure to COVID-19 during treatment planning and delivery.

a. With regards to the issue of PPE, provide adequate protective equipment, including N-95 masks for therapists and other staff who are in close contact with patients, especially during relatively high-risk portions of the radiosurgery cycle such as stereotactic frame application/removal, mask fabrication and positioning for radiosurgery treatment.

b. Favor mask immobilization rather than frames, in situations, such as for multiple brain metastases, in which either might be appropriate. Although neither are risk free in terms of closeness of contact between members of the radiosurgery team and patient, arguably there is less intensity of exposure during mask fabrication and positioning than there is in the process of frame placement, positioning for treatment, and removal.

c. Consider immobilization devices to be contaminated, particularly for cranial radiosurgery, irrespective of radiosurgery treatment platform. Stereotactic frames are in close proximity to nasal and oral secretions. Face masks are in direct contact with the nose and mouth. Even the fiducial marker stickers placed on the nose for Gamma Knife frameless radiosurgery should be managed as a contaminated item.

4. Minimize number of fractions. Another strategy to minimize contact and help with decreasing the flow of individuals into the hospital is to focus on minimizing the number of fractions in our treatment plans whenever feasible. As a result of the COVID-19 outbreak, our center has been reducing the number of fractions for fractionated stereotactic radiosurgery (SRS) and using frameless options when feasible.

5. Minimize trips for the patient to the medical center. Coordinate care so that as many tasks are arranged on the same day as possible for each patient; these tasks include the clinician visit (if unable to be done virtually), CT simulation scan, and treatment planning magnetic resonance imaging (MRI) all to be completed in one visit to minimize the hospital trips and unnecessary exposure.

6. Defer treatment of radiographically stable patients. Sometimes, after discussion of treatment with radiosurgery, a treatment planning MRI reveals stable metastases. We have proposed observing those lesions, rather than proceeding with treatment, especially if the patient is on or may be started on a systemic regimen deemed likely to be effective in the brain. We also discuss observing stable lesions in certain cases when this is a safe option for the patient.

7. Use systemic therapy as a temporizing measure, especially if indicated for systemic disease.

8. Minimize use of corticosteroids due to risk of inciting or exacerbating immune suppression.

9. In situations in which surgery rather than radiosurgery might be favored but not absolutely urgent (e.g., relatively large tumor, mild/moderate neurological symptoms), consider a trial of upfront radiosurgery with a plan for surgery as salvage treatment.

10. Consider single fraction radiosurgery for multiple brain metastases rather than whole brain irradiation, if clinically reasonable, as the latter requires 10-15 sessions, while the former can be accomplished in one visit. If the number of metastases is too large for treatment with single fraction radiosurgery, and a few dominant metastases can be identified, consider single fraction treatment of those lesions, with salvage additional radiosurgery (or whole brain radiation) in the future.

## Conclusions

The COVID-19 pandemic is affecting all aspects of the healthcare ecosystem, including administration of radiosurgery. Innovative solutions will be driven by increased interdisciplinary and global dialogue. Focusing on emerging data from centers where the epidemiological peak has already occurred will be helpful in refining treatment guidelines to optimize care in this setting. Overall, our patients will continue to benefit from efforts to minimize unnecessary exposure to each other and health care workers. Telemedicine is well suited to many facets of the workflow for stereotactic radiosurgery and will play a new prominent role in these efforts.
